# High-performance achromatic flat lens with high NA

**DOI:** 10.1038/s41377-025-01943-6

**Published:** 2025-08-27

**Authors:** Yifeng Shao, Paul Urbach

**Affiliations:** Optics Research, Delft University of Technology, Delft, Netherlands

**Keywords:** Metamaterials, Nanophotonics and plasmonics

## Abstract

A new strategy has been presented to overcome the long-term dilemma of simultaneously achieving high numerical aperture, large aperture size, and broadband achromatism of flat lenses. A stepwise phase dispersion compensation (SPDC) layer is introduced as a substrate on which the meta-atoms are positioned.

Chester Moor Hall invented the achromatic lens in 1729. In modern information technology, new types of achromatic flat lenses for miniaturized, ultracompact, and lightweight optical systems^1,2^ are desired. By using subwavelength-scale meta-atoms, phase dispersion control is possible^3,4^. A proper arrangement of meta-atoms can achieve achromatic focusing and imaging, enabling the design of ultrathin achromatic flat lenses^5,6^. However, the ability of these meta-atoms to control phase dispersion is limited, making it difficult to achieve simultaneously a high numerical aperture (NA), large aperture size, and broadband achromatic performance^7,8^.

Researchers have explored various approaches to solve these issues. Pioneering efforts focused on enhancing the phase dispersion control capability of individual meta-atoms to achieve achromatic focusing^9,10^. Subsequently, it was discovered that using asymptotic fitting methods could further improve the performance of achromatic focusing^11^. Additionally, multi-level diffractive lenses have been demonstrated to operate over a wide wavelength range with large aperture sizes^12^. Hybrid or multilayer lenses have also been proposed to enhance imaging performance by combining multiple optical elements or layers^13,14^. For instance, hybrid lenses fabricated via laser writing have been proposed to modulate phase dispersion and achieve wide bandwidth achromatic focusing^15^. These approaches aim at finding an ultimate solution for creating high-performance achromatic flat lenses. However, the challenge of simultaneously achieving high numerical aperture, large aperture size, and broadband achromatic performance remained an unsolved problem.

The research team led by Prof. Xue-Hua Wang from Sun Yat-sen University in China proposed a novel method^16^. They applied meta-atoms on a stepwise phase dispersion compensation (SPDC) layer. In this way, basically any desired radially varying phase can be realized by increasing the thickness difference of the rings of the SPDC layer. In this way, the phase dispersion of the meta-atoms across a wide spectrum can be compensated. Thus, the limitations imposed by the maximum achievable phase dispersion of the meta-atom library are overcome.

The authors demonstrated the effectiveness of their strategy with the example shown in Fig. 1. Given a meta-atom library with a maximum phase dispersion of only 2.2π, a conventional achromatic metalens using meta-atoms for a bandwidth of 350 nm can achieve only a relatively low NA of 0.56 and a small radius of 6.6 μm. However, by incorporating an appropriate SPDC layer, the authors realized a flat lens with the same achromatic bandwidth and focal length but with a significantly higher NA of 0.9 and a larger radius of 20.1 μm. To construct an achromatic metalens with the same performance using only meta-atoms, the maximum phase dispersion tuning range of the meta-atoms would need to be increased by a factor of six, which is highly challenging. This study confirms that the SPDC layer effectively enhances the focusing performance of the proposed achromatic flat lenses.

**Fig. 1:**
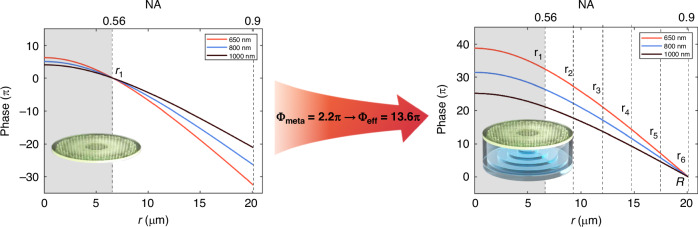
Left: Phase profiles of a conventional achromatic flat lens ranging for wavelength from 650 nm to 1000 nm. Due to the limited phase dispersion of the meta-atoms, the achievable NA and aperture size are restricted. Right: By incorporating the SPDC layer, the achievable effective phase dispersion is significantly increased, thereby overcoming the problem of realizing at the same time broadband, high NA, and large aperture size in an achromatic flat lens

Designs of broadband achromatic flat lenses with SPDC layer were simulated with the FDTD method and were fabricated. The PSF was measured with a high NA microscope and compared to simulations using the Richards–Wolf vectorial diffraction theory. Near diffraction-limited imaging was demonstrated. The multiplex of the meta-atoms can be repeated indefinitely so that large aperture size achromatic flat lenses can be achieved. It would be interesting to quantify the performance of the lens by measuring aberration coefficients. This is rarely done by researchers working in the field of meta lenses, which is a pity because aberration coefficients give the best information about the quality of a lens.

In summary, the authors have developed and demonstrated a new design of an achromatic flat metalens with very high NA and large aperture size.
